# Association of admission serum levels of vitamin D, calcium, Phosphate, magnesium and parathormone with clinical outcomes in neurosurgical ICU patients

**DOI:** 10.1038/s41598-018-21177-4

**Published:** 2018-02-14

**Authors:** Seyed Hossein Ardehali, Salman Dehghan, Ahmad Reza Baghestani, Aynaz Velayati, Zahra Vahdat Shariatpanahi

**Affiliations:** 1 Department of Anesthesiology and Critical Care, Shohada Tajrish Hospital, Shahid Beheshti University of Medical Sciences, Tehran, Iran; 2 National Nutrition and Food Technology Research Institute, Faculty of Nutrition and Food Technology, Shahid Beheshti University of Medical Sciences, Tehran, Iran; 3 Department of Biostatistics, School of Public Health, Shahid Beheshti University of Medical Sciences, Tehran, Iran

## Abstract

To evaluate the association of admission serum levels of 25(OH)D, parathormone and the related electrolytes with severity of illness and clinical outcomes in neurosurgical critically ill patients, serum levels of 25(OH)D, parathormone, calcium, magnesium, and phosphate, along with APACHE II score were measured for 210 patients upon admission. Mean serum 25(OH)D was 21.1 ± 7.4 ng/mL. 25(OH)D deficiency (less than 20 ng/dL) and elevated serum parathormone level were found in 47.6% and 38% of patients respectively. Hypocalcaemia, hypophosphatemia, hypomagnesaemia and hypermagnesaemia were found in 29.5%, %63.8, 41.9% and 27.6% of patients respectively. The APACHE II score was significantly correlated with serum levels of 25(OH)D, parathormone, calcium, and phosphate. Multivariate regression analysis adjusted by other risk factors showed that among all clinical outcomes, admission hypovitaminosis D was associated with longer duration of ICU stay and a high admission of parathormone was associated with in ICU mortality. We concluded that disorders of admission serum levels of 25(OH)D, parathormone, calcium, magnesium, and phosphate are related to the presence of multiple causal factors such as severity of disease and are not independently associated with clinical outcomes. Most often they are normalize spontaneously with resolution of the disease process.

## Introduction

Vitamin D deficiency has been reported to be widely prevalent in critically ill patients. The prevalence of vitamin D deficiency in the intensive care unit (ICU) has ranged from 44.2 to 82.6%^1–9^. Vitamin D has a wide range of functions in extrascletal tissues and its deficiency has been associated with chronic diseases^1^. It seems that hypovitaminosis D is responsible for adverse clinical outcomes in critically ill by similar mechanisms to those in chronic conditions. Several studies have evaluated the effect of admission hypovitaminosis D on clinical outcomes in septic^7^, medical^2^, mixed^8^ surgical^3–6^ and neurological ICU patients^9^. In addition it has been reported that concomitant high serum parathyroid hormone (PTH) with hypovitaminosis D is associated with higher mortality rate^10^.

On the other hand, critically ill patients usually have electrolyte disorders on admission in the ICU. Changes in serum calcium, phosphate and magnesium, the electrolytes related to vitamin D as frequently electrolyte disorders seen on admission in the ICU and their affects on clinical outcomes are under debate.

Admission hypocalcaemia is a common electrolyte disorder in the critically ill patients with prevalence of 50–88%. It is usually in response to inflammation^11^. Some studies have shown that hypocalcaemia in critically ill patients is associated with increased mortality^12–14^ while others have not find this association^15,16^. The prevalence of hypophosphatemia on admission in the ICU is 13–80%. However, there is controversy over its correlation with clinical outcomes^17^. Studies have shown diverse results in regard to admission serum phosphate level and clinical outcomes^18–22^. The prevalence of admission hypomagnesaemia in the ICU is 18–65%. There are many studies investigating the correlation between admission hypomagnesaemia and clinical outcomes in ICU with conflicting results^23–33^.

Since the relation between PTH, vitamin D, calcium, phosphate and magnesium has been documented, the aim of this study was to determine their serum levels on admission in neurosurgical ICU patients and their association with clinical outcomes.

## Methods

This was a prospective cross-sectional study conducted in 210 patients admitted to the neurosurgical intensive care unit in a university hospital between April 2015 and March 2016. The patients or their surrogates completed the written informed consent. The study was approved by the Shahid Beheshti University ethics committee and have therefore been performed in accordance with the ethical standards laid down in the 1964 Declaration of Helsinki and its later amendments. All consecutive patients 18 years and older and who were expected to require more than 48 hours in the ICU and were enrolled within the first 24 hours of the ICU admission, were deemed eligible to participate. Exclusion criteria included breast feeding, pregnancy, impaired renal function on admission, severe liver disease, diseases of parathyroid and subjects who were transferred from other ICUs.

## Data Collection

Serum 25(OH)D, PTH, total calcium, phosphate, magnesium, albumin and total protein, were measured within 24 hours of ICU admission. Necessary variables were recorded to calculate the acute physiology and chronic health evaluation II (APACHE II) score on the first day of admission. Occurrence of organ failure was monitored during hospitalization. Each patient was evaluated daily for cardiovascular failure, central nervous system failure, coagulation failure, hepatic failure and renal failure. Mean sequential organ failure assessment (SOFA) score was used to determine the extent of a person’s organ function. Other clinical outcomes were rate of mortality, use of mechanical ventilation, and hospital length of stay.

Definition of Vitamin D deficiency: 25 (OH) D is considered the best available parameter for the definition of vitamin D status, since it reflects vitamin D body stores better than other vitamin D metabolites. To date, there is no consensus regarding normal vitamin D levels and the definition of vitamin D deficiency in critically ill patients. We categorized 25 (OH) D levels as follows: severe deficiency with serum levels less than 10 ng/dL, mild deficiency with serum levels as 10–20 ng/dL, insufficiency with serum levels as 20–30 ng/dL and normality with serum levels more than 30 ng/dL^34,35^.

Serum calcium, magnesium, phosphate and PTH were measured on the day of admission. Hypocalcaemia and hyperkalemia were defined as serum calcium levels less than 8.5 mg/dL and more than 10.6 mg/dL respectively. Corrected calcium was calculated by measured total calcium + (0.8 × (4.0 − (albumin))). Hypophosphatemia and hypophosphatemia were defined as serum phosphate levels less than 3.5 mg/dL and more than 4.5 mg/dL respectively. Hypomagnesaemia and hypermagnesaemia were defined as serum magnesium levels less than 1.7 mg/dL and more than 2.1 mg/dL respectively. Elevated PTH level was defined as a serum PTH level more than 65 pg/mL^36^.

Statistical analysis: Data were analyzed using the SPSS Version 21 software. Subjects’ characteristics are reported as mean ± SD and median, interquartile range for continuous variables and in frequencies and percentages for categorical ones.

Differences between variables in categorical groups of vitamin D were evaluated with the one-way analysis of variance (ANOVA), Kruskal-Wallis, the chi-square or Fisher Exact test. Regression analysis was used to estimate the relationship between serum vitamin D, PTH, calcium, magnesium and phosphate levels with clinical outcomes (mortality, length of stay, duration of mechanical ventilation and organ failure). In this model length of stay (LOS) in the ICU, SOFA score for organ failure and duration of mechanical ventilation were categorized by their median to two groups. Any covariate associated with the response variables (P < 0.05) in univariate analysis, retained in the final model or multivariate logistic regression. Furthermore variables that could have a clinical significance, retained in the final model. P-values less than 0.05 were considered significant.

## Results

A total of 250 patients participated in the study. Forty were excluded due to discharge or death before the second admission day and missing data. Therefore, 210 patients (98 females and 112 males) were included in the study. Admission diagnosis was brain tumor surgery (n = 108, 51.4%), spinal tumor surgery (n = 8, 3.8%), intracranial hemorrhage (n = 64, 30.4%), vertebral fixation (n = 26, 12.3%) and chiari malformation (n = 4, 1.9%).

Median LOS in the ICU was 6 days in total population with minimum and maximum of 3 and 56 days respectively. Organ failure was assessed by the SOFA score. Median SOFA score was 5 in total population with minimum and maximum of 2 and 12 score respectively. One hundred eight (51.4%) patients were under mechanical ventilation. Median duration of mechanical ventilation was 6 days with minimum and maximum of 2 and 51 days respectively. Rate of mortality was 27.6% in total population (n = 58).

### Serum level of 25 (OH) vitamin D and outcomes

Table 1 shows baseline and clinical characteristics of the patients according to four categorized groups of serum vitamin D. Mean serum 25 (OH) D was 21.1 ± 7.4 ng/mL in total population. Sixteen (7.6%) patients were with severe 25 (OH) D deficiency, 84(40.1%) with mild 25(OH) deficiency, 76 (36.1%) with 25(OH) insufficiency and 34 (16.1%) were in normal range and their difference was significant.

**Table 1:** Characteristics of patients based on 25-hydroxyvitamin D categorized groups.

	Deficiency Severe (n = 16)	Deficiency Mild (n = 84)	Insufficiency (n = 76)	Normal (n = 34)	P value
Age (year)	47 ± 16^ab^	51 ± 12^a^	42 ± 14^b^	42 ± 15^ab^	0.025*
Sex (n. %)					0.63***
Male	10 (62.5%)	48 (57.1%)	48 (63.2%)	18 (52.9%)	
Female	6 (7.5%)	36 (42.9%)	28 (36.8%)	16 (47.1%)	
APACHE II,	12.75 ± 3.7^a^	11.33 ± 4.54^ab^	8.42 ± 3.86^bc^	8.41 ± 3.29^c^	0.002*
Albumin (g/dL)	2.9 ± 0.55^a^	3.1 ± 0.5^ab^	3.4 ± 0.35^b^	3.3 ± 0.87^ab^	0.02*
25(OH)D (ng/dL)	8.6 ± 0.6^a^	16.08 ± 2.7^b^	24.04 ± 2.5^c^	33.18 ± 2.3^d^	0.001*
Calcium (mg/dL)	8.5 ± 0.45	8.7 ± 0.75	8.9 ± 0.7	8.8 ± 0.81	0.40*
Magnesium (mg/dL)	1.75 ± 0.19	1.87 ± 0.32	1.97 ± 0.41	1.78 ± 0.31	0.22*
Phosphate (mg/dL)	3.4 ± 1.1	3.06 ± 0.73	3.4 ± 0.78	3.3 ± 0.87	0.23*
PTH (pg/mL)	122.4 ± 32.7^a^	77.3 ± 48.7^b^	56.7 ± 33.1^b^	48.1 ± 31.7^b^	0.001*
Blood glucose (g/dL)	138.07 (87.2)	129.6 (38.6)	118.6 (39.9)	118 (37.3)	0.088*
Energy intake (%)	56.1 ± 27.7	68.8 ± 21.9	61.9 ± 25.3	56.1 ± 21.7	0.19*
LOS^1^	17.5 (25.5)^a^	13 (15)^a^	4 (2)^b^	3 (5.1)^b^	0.001**
SOFA^2^	8 (3)^a^	5(4)^ac^	4 (4.25)^c^	5 (2.5)^c^	0.006**
MV^3^ (n, %)	16 (7.6%)^a^	60 (28.6%)^b^	24 (12.5%)^a^	8 (3.9%)^a^	0.04***
MV Duration	19.6 ± 10.3^a^	12.7 ± 9.7^b^	4.1 ± 1.58^c^	1.93 ± 0.94^c^	0.001**
Mortality (n, %)	12 (20.7%)^a^	34 (58.6%)^ab^	10 (17.2%)^bc^	2 (3.4%)^c^	0.001***

^*^One-way ANOVA (mean ± SD), **Man-Whitney (median, IQ), ***Chi square.

^1^Length of stay, ^2^Sequentional organ failure assessment, ^3^Mechanical ventilation. Values with different superscript letters are significantly different.

Median length of stay in the ICU was significantly higher in the severe deficiency and mild deficiency groups compared to the insufficiency and normal groups (Table 1). Result of univariate logistic regression analysis with the LOS as dependent variable and serum 25(OH)D as independent variable showed that with each point increase in serum 25(OH)D, risk of hospitalization more than 6 days is decreased by 20% (Table 2).

**Table 2:** Logistic regression for clinical outcomes in 210 patients according to serum 25(OH)D and parathormone level.

	OR	95% CI	P value	Adjusted OR	95% CI	P value
**Mortality**						
25(OH)D	0.85	0.78–0.92	0.001			
PTH	1.03	1.01–1.04	0.001	1.02	1.002–1.040	0.03
Calcium	0.48	0.25–0.92	0.029			
Phosphate	0.87	0.51–1.4	0.600			
Hypomagnesaemia	1.15	0.38–3.45	0.852			
hypermagnesaemia	0.85	0.30–2.38	0.760			
**LOS** ^**1**^ **>6 days**						
25(OH)D	0.80	0.74–0.88	0.001	0.89	0.79–0.99	0.04
PTH	1.01	1.00–1.02	0.001			
Calcium	0.58	0.33–1.20	0.062			
Phosphate	0.67	0.40–1.11	0.128			
Hypomagnesaemia	2.04	0.73–5.73	0.174			
hypermagnesaemia	1.32	0.51–3.40	0.563			
**SOFA** ^**2**^ **>7**						
25(OH)D	0.92	0.87–0.98	0.001			
PTH	1.01	1.007–1.02	0.001			
Calcium	0.65	0.36–1.10	0.156			
Phosphate	0.82	0.49–1.30	0.596			
Hypomagnesaemia	0.75	0.25–2.17	0.455			
hypermagnesaemia	0.37	0.13–1.05	0.060			
**MV** ^**3**^						
25(OH)D	0.88	0.79–0.97	0.013			
PTH	1.01	0.90–1.02	0.114			
Calcium	0.58	0.25–1.30	0.206			
Phosphate	0.73	0.38–1.40	0.350			
Hypomagnesaemia	1.35	0. 43–4.19	0.600			
Hypomagnesaemia	0.29	0.41–1.62	0.631			

^1^Length of stay; ^2^Sequential Organ Failure Assessment Score; ^3^Duration of mechanical ventilation.

The mean serum level of 25 (OH) D was 23.08 ± 6.9 ng/dL in dead patients and 16.15 ± 6.1 ng/dL in alive patients (P = 0.001). Rate of mortality was significantly higher in the severe deficiency group compared to the insufficiency and normal groups, as well as in the mild deficiency group compared to the normal group (Table 1). Result of univariate logistic regression analysis with mortality as dependant variable and serum 25(OH)D as independent variable showed that with each point increase in serum 25(OH)D, risk of mortality decreased by 15% (Table 2).

The mean serum level of 25 (OH) D was 17.98 ± 7.1 ng/dL in ventilated patients and 24.53 ± 6.2 ng/dL in non-ventilated patients (P = 0.001). All patients in the severe 25(OH) D group were under mechanical ventilation and the mean duration of mechanical ventilation was significantly higher in the severe deficiency and mild deficiency groups compared to the insufficiency and normal groups (Table 1). Result of univariate logistic regression analysis with duration of mechanical ventilation as the dependant variable and serum 25(OH)D as the independent variable showed that with each point increase in serum 25(OH)D, risk of ventilation more than 6 days decreased by 12% (Table 2).

Median SOFA score was significantly higher in the severe deficiency group compared to the mild deficiency, insufficiency and normal groups (Table 1). Result of univariate logistic regression analysis with the SOFA score as the dependant variable and serum 25(OH)D as the independent variable showed that with each point increase in serum 25(OH)D, risk of SOFA score more than 5 decreased by 8% (Table 2).

A multivariate logistic regression analysis with serum 25(OH)D as the regressor and each clinical outcome as the dependant variable was separately built (adjusted by APACHE II score, serum albumin, PTH, calcium, phosphate, magnesium, energy intake, age and sex). In this regard, results showed that with each point increase in serum 25(OH) level, risk of hospitalization more than 6 days decreased by 11% whereas serum level of 25(OH)D was not an independent variable for mortality, mechanical ventilation and organ failure (Table 2).

Results of Pearson correlation showed that serum vitamin D was significantly correlated with PTH, albumin, total protein and APACHE II (r = −0.37, P < 0.001; r = 0.23, P = 0.01; r = 0.2, P = 0.03; r = −0.37, P < 0.001 respectively).

### Serum level of calcium and outcomes

The mean total serum calcium corrected for albumin was 8.85 ± 0.73 mg/dL with minimum value of 7.20 and maximum value of 10.50. Sixty two patients (29.5%) had low serum calcium (lower than 8.5 mg/dL). Median length of stay in the ICU and median SOFA score were not different between the calcium deficient group and the normal group (P = 0.2, P = 0.8 respectively).

The mean serum level of calcium was 8.67 ± 0.67 mg/dL in ventilated patients and 9.03 ± 0.75 mg/dL in non-ventilated patients (P = 0.5). Results of univariate logistic regression analysis with each clinical outcome separately (LOS, SOFA score and duration of mechanical ventilation) as the dependant variable and serum calcium level as the independent variable showed that there was no relation between serum calcium level and clinical outcomes (Table 2).

The mean serum level of calcium was 8.95 ± 0.74 mg/dL in dead patients and 8.59 ± 0.63 mg/dL in alive patients (P = 0.4).

Results of Pearson correlation showed that serum calcium was significantly correlated with serum magnesium, albumin, total protein and APACHE II (r = 0.19, P = 0.04; r = 0.35, P < 0.001; r = 0.4, P < 0.001; r = −0.32, P = 0.001 respectively).

Result of univariate logistic regression analysis with mortality as the dependant variable and serum calcium level as the independent variable showed that with each point increase in serum calcium level, risk of mortality decreased by 13% (Table 2), but in multivariate logistic regression model adjusted by APACHE II score, serum albumin, PTH, 25(OH) D, phosphate, magnesium, energy intake, age and sex, this relation was not observed (Table 2).

### Serum level of phosphate and outcomes

The mean serum phosphate level was 3.26 ± 0.81 mg/dL with minimum value of 1.40 and maximum value of 5.5. Hypophosphatemia with serum phosphate level lower than <3.5 mg/dL was seen in 63.8% (n = 134) of patients. Hyperphosphatemia was seen only in one patient with serum level of 5.5 mg/dL. We did not consider this patient in the statistical analysis. Median length of stay in the ICU and median SOFA score was not different between the phosphate deficient group and the normal group (P = 0.8, P = 0.8 respectively). The mean serum level of phosphate was 3.19 ± 0.77 mg/dL in dead patients and 3.28 ± 0.83 mg/dL in alive patients (P = 0.8). The mean serum level of phosphate was 3.2 ± 0.87 mg/dL in ventilated patients and 3.32 ± 0.75 mg/dL in nonventilated patients (P = 0.4).

Results of Pearson correlation showed that serum phosphate level was significantly correlated with albumin, total protein and APACHE II (r = 0.21, P = 0.02; r = 0.2, P = 0.02; r = −0.19, P = 0.05 respectively).

Result of univariate logistic regression analysis with each clinical outcome (LOS, SOFA score, duration of mechanical ventilation and mortality) as the dependant variable and serum phosphate level as the independent variable showed that there was no relation between serum phosphate level and clinical outcomes (Table 2).

### Serum level of magnesium and outcomes

The mean serum magnesium level was 1.88 ± 0.35 mg/dL with minimum value of 0.90 mg/dL and maximum value of 3.5 mg/dL. A total of 88 (41.9%) patients had elevated magnesium level (serum magnesium level >2.1 mg/dL) and 58 (27.6%) patients had reduced magnesium level (serum magnesium level <1.7 mg/dL). Median length of stay in the ICU and median SOFA score was not different between the hypomagnesemic, hypermagnesemic and normal groups (P = 0.69, P = 0.08 respectively). The mean serum level of magnesium was 1.86 ± 0.38 mg/dL in dead patients and 1.89 ± 0.38 mg/dL in alive patients (P = 0.46). The mean serum level of magnesium was 1.85 ± 0.37 mg/dL in ventilated patients and 1.92 ± 0.33 mg/dL in nonventilated patients (P = 0.71).

Result of univariate logistic regression analysis with categorized serum magnesium level as the independent variable and each clinical outcome (LOS, SOFA score, duration of mechanical ventilation and mortality) as the dependent variable showed that there was no relation between serum magnesium level and clinical outcomes (Table 2).

### Serum level of parathormone and outcomes

The mean serum PTH level was 68.5 ± 43.9 pg/mL with minimum value of 17.9 pg/mL and maximum value of 178.7 pg/mL. A total of 84 (38%) patients had elevated PTH level (serum PTH level >65 pg/mL). Result of univariate logistic regression analysis with serum PTH level as the independent variable and each clinical outcome (LOS, SOFA score, duration of mechanical ventilation and mortality) as the dependant variable showed that with increasing serum PTH level risk of mortality, LOS in the ICU and SOFA score increased significantly. In multivariate logistic regression model (adjusted by APACHE II score, serum albumin, calcium, 25(OH)D, phosphate, magnesium, age, sex, and calorie intake), only serum PTH was an independent variable for mortality (Table 2).

Secondary hyperparathyroidism was defined as elevated serum PTH level associated with serum 25(OH) level lower than 30 ng/mL and/or serum calcium level lower than 8.5 mg/dL. Secondary hyperparathyroidism was seen in 51.6% of hypocalcemic and in 45.4% of vitamin D insufficient/deficient patients. Table 3 shows that PTH responders have higher APACHE II score, SOFA score, LOS in ICU, ventilator dependency, and mortality compared to non- responders (Table 3).

**Table 3:** Characteristics of patients based on serum PTH.

	Hyperparathyroidism group (n = 84)	Non hyperparathyroidism group (n = 126)	P value
APACHE II,	11.88 ± 4.26	8.81 ± 4.15	0.001*
25(OH)D (ng/dL)	16.55 ± 5.21	20.75 ± 5.21	0.001*
Calcium (mg/dL)	8.64 ± 0.63	9.03 ± 0.73	0.01*
Magnesium (mg/dL)	1.90 ± 0.39	1.91 ± 0.34	0.94*
Phosphate (mg/dL)	3.13 ± 0.78	3.34 ± 0.82	0.22*
PTH (pg/mL)	113.9 ± 32.59	38.06 ± 14.10	0.001*
LOS^1^	13 (6–28)	5 (4–8)	0.001**
SOFA^2^	7 (5–9)	4 (3–6)	0.001**
MV^3^ (n, %)	68 (68%)	32 (32%)	0.04***
Mortality (n, %)	47 (81%)	11 (19%)	0.001***

^*^T test (mean ± SD), **Man-Whitney (median, IQ), ***Chi square.

^1^Length of stay, ^2^Sequentional organ failure assessment, ^3^Mechanical ventilation.

Results of Pearson correlation showed that PTH had was significantly correlated with albumin, vitamin D and APACHE II (r = −0.29, P = 0.003; r = −0.37, P < 0.001; r = 0.35, P < 0.001 respectively).

## Discussion

Our study showed that among all clinical outcomes, admission hypovitaminosis D was associated with longer duration of stay in the ICU and a high admission level of parathormone was associated with mortality in the ICU. Furthermore, PTH responders had higher APACHE II score, SOFA score, LOS in the ICU, ventilator dependency, and mortality compared to non- responder patients. We did not find any correlation between admission serum calcium, magnesium and phosphate level with clinical outcomes. There was a negative correlation between APACHE II score, serum calcium, phosphate, and vitamin D and a positive correlation with serum PTH.

### Serum level of vitamin D and outcomes

We found that 47.7% of patients had vitamin D deficiency and hypovitaminosis D was an independent risk factor for longer duration of stay in the ICU. Many retrospective and prospective studies have evaluated the effect of admission serum vitamin D level on clinical outcomes of medical, surgical, cardiac, mixed and traumatic critically ill patients. Some reported that hypovitaminosis D was associated with increased mortality^2,4,5,8^, longer ICU stay^3–5^ and greater need for mechanical ventilation^6^, whereas some results did not show this association concerning mortality in the ICU^9^, duration of stay in ICU^2,8,9^, organ failure^7^ and duration of mechanical ventilation^2,8,9^. In one study conducted in the neurocritical care patients, vitamin D deficiency was associated with higher in-hospital mortality in the subset of patients admitted on an emergency basis and this association was not seen in the total population (9). Several factors may contribute to vitamin D deficiency in critically ill patients with prolonged hospitalization including lack of exposure to sunlight, malnutrition, decreased renal hydroxylation and increased tissue conversion of 25(OH)D_3_ to 1,25(OH)_2_D_3_ however the reasons for the reduced serum level of vitamin D in the first day of hospitalization may be the reduced serum level of albumin or vitamin D binding protein, or receiving intravenous volumes in order to correct hypovolemia or hypotension^37^. The poor clinical outcomes seen in critically ill patients with admission hypovitaminosis D may be related to severity of the underlying disease. The extent of hypoalbinemia is related to the degree of inflammation and severity of acute illness and also the extent of volume replacement is related to the severity of disease^37^ as we found an inverse correlation between admission serum vitamin D and APACHE II score as well as a direct correlation between admission serum vitamin D and serum albumin. Also it has been reported that novel vitamin D3 hydroxyderivatives resulting from the action of cytochrome P450 side chain cleavage, have anti-proliferative, prodifferentiation and anti-inflammatory activities and measuring their serum levels may be necessary to fully assess vitamin defciency or suffciency, as opposed to a single measurement of 25(OH)D3^38–40^.

### Serum level of calcium and outcomes

The prevalence of hypocalcaemia was 29.5%. We did not find any correlation between admission serum calcium level and clinical outcomes, however, we found an inverse correlation between admission hypocalcaemia and APACHE II score. Various studies have evaluated the effect of admission serum calcium level on clinical outcomes of medical, surgical, mixed and traumatic critically ill patients with diverse results. Results of some studies reported by comparing means, have shown that severe and moderate hypocalcaemia (either total or ionized) is associated with increased mortality and worse clinical outcomes in the critically ill patients^12–14^. In our study most of the patients had mild hypocalcaemia. It has been reported that mild hypocalcaemia has protective effects in critically ill patients and its correction is not recommended^41^. Results of some other studies using multivariate methods are consistent with our findings as they did not find any association between hypocalcaemia and clinical outcomes^15,16^. Abnormal values of admission serum calcium level in the ICU are common which are not the result of an underlying disease in most cases. Hypocalcaemia is a marker of disease severity and inflammation in the ICU as we found an inverse correlation between admission serum calcium and APACHE II score with resolution of the primary disease, hypocalcaemia will normalize^11^.

### Serum level of phosphate and outcomes

The prevalence of hypophosphatemia was 63.8%. We did not find any correlation between admission serum phosphate level and clinical outcomes. Some studies have shown that hypophosphatemia in critically ill patients is associated with increased mortality^18–20^, while others did not find this association^21,22^. There are many factors responsible for the occurrence of hypophosphatemia in the ICU including sepsis, trauma, volume expansion, refeeding syndrome, acid-base disorders, medications, major surgery, head trauma and renal replacement therapyand it is unclear whether correcting admission hypophosphatemia improves outcomes in critically ill patients.

### Serum level of magnesium and outcomes

The prevalence of hypomagnesaemia and hypermagnesaemia were 27.6% and 41.9% respectively. We did not find any association between admission serum magnesium and clinical outcomes. Several studies have evaluated the effect of admission serum magnesium level on clinical outcomes in medical, surgical, traumatic and mixed critically ill patients. Some have shown that hypomagnesaemia (either total or ionized) is associated with increased mortality^23–30^, longer ICU stay^25,26^, greater need for mechanical ventilation^24,26,30^, higher SOFA scores^28^ while others did not find this association^31–33^. Results of some were reported by comparing means and without performing regression analysis^23–26,30^. Several factors are responsible for changes of admission serum magnesium. Some alter magnesium transport in the loop of Henle including PTH, calcitonin, glucagon, arginine vasopressin, and the beta-adrenergic agonists. Hypoalbinemia, nutrition, use of diuretics, metabolic acidosis, hypokalemia, phosphate depletion, volume expansion and aminoglycosides are other etiologies of hypomagnesaemia in critically ill patients^42^.

Moreover there is no clear evidence that magnesium supplementation improves outcomes in critically ill patients^43^.

### Serum level of parathormone and outcomes

Our study indicated that with increasing PTH level, risk of stay in the ICU increased by 2%. PTH is a marker of inflammation and disease severity. It has been reported that high PTH levels are associated with higher mortality rate^44^. With increasing disease severity and inflammation, serum PTH levels will increase more^45^. It is the reason for the existing of a significant correlation between admission serum PTH and APACHE II score in our study.

On the other hand, we found that PTH responders had higher LOS in the ICU, ventilator dependency, and mortality compared to non- responder patients. This might be due to higher APACHE II score and therefore, severity of disease in responders compared to non- responders. However, previous studies had conflicting results. Some showed that there was no difference between the PTH responders and non-responders in regard to clinical outcomes^46^, while others showed higher mortality rate in the PTH responders^10^.

## Conclusion

It seems that changes in serum vitamin D, calcium and PTH on admission are the result of inflammation related to severity of diseases. Values of serum PTH, calcium and vitamin D will return to actual values with remission of inflammation. On the other hand, admission electrolyte disorders due to the presence of multiple causal factors are among the most common clinical problems in the critically ill patients. The vast majority of these patients do not have an underlying related electrolyte disease and most often the electrolyte disorders normalize spontaneously with resolution of the primary disease process. Conducting studies with measuring these parameters at other times of hospitalization is recommended. Moreover further studies to assay the effect of correcting these disorders on clinical outcomes are required.
